# Cephalometric Analysis of Maxillary Sinus for Gender Determination Using Lateral Cephalogram: A Novel Retrospective Study in the North Indian Population

**DOI:** 10.7759/cureus.66030

**Published:** 2024-08-02

**Authors:** Ajay Kumar, Shailendra S Rana, Shreyas Gupta, Apurba Patra

**Affiliations:** 1 Forensic Medicine, All India Institute of Medical Sciences, Bathinda, Bathinda, IND; 2 Dentistry, All India Institute of Medical Sciences, Bathinda, Bathinda, IND; 3 Anatomy, All India Institute of Medical Sciences, Bathinda, Bathinda, IND

**Keywords:** sexual dimorphism, forensic anthropology, lateral cephalogram, maxillary sinus, sex determination

## Abstract

Background: Underlying disorders of the maxillary sinus (MS), including a history of sinus surgeries, chronic sinusitis, or congenital anomalies can potentially impact sinus function and structure, necessitating careful evaluation and management. Moreover, intact sinuses are crucial in gender determination in forensic anthropology. The present study was undertaken to check the accuracy and reliability of MS in gender determination using morphometric parameters.

Materials and methods: This retrospective study was carried out on 74 lateral cephalograms (37 males and 37 females) aged between 18 to 50 years from the North Indian population. The MS area was measured using a NewTom CBCT machine (NewTom, Imola, Italy) with slicer software. The anatomical landmarks for the sinus were identified, and the area was calculated in square millimeters (mm^2^).

Results: In terms of surface area, females had a mean of 13,210.40 mm^2^ with a standard error of 713.46. Males, however, exhibited a higher mean surface area of 18,713.82 mm^2^, but with a significantly larger standard error of 3,371.70. The difference in MS area between males and females was statistically significant (p<0.01). In the receiver operating characteristic (ROC) curve, the area under the curve (AUC) was 0.77, suggesting good discriminative ability.

Conclusion: The MS area on lateral cephalograms shows significant sexual dimorphism. Overall, the findings suggest that the MS surface area can be a useful anatomical feature for distinguishing between male and female North Indian subjects, given the statistically significant difference and the good discriminative performance indicated by the ROC curve analysis.

## Introduction

The morphological and radiological evaluations of paranasal sinuses, specifically maxillary sinus (MS) provide detailed informa­tion on the anatomy and pathology of the region [1]. MS is the largest of the paranasal sinuses, that reaches its final dimensions by the end of the second decade of life. The morphology of MS (size-shape) and scaling on mucosal surface area dynamics may have implications for hypotheses that structurally link MS morphology to craniofacial ontogeny [2]. In forensic science, gender determination is a crucial step in establishing identification. According to Kanthem et al., MS exhibits anatomic variability between the genders and its dimensions, especially the volume, which is crucial in studying sexual dimorphism [3]. Traditionally, radiological findings are crucial in forensic identification; however, accuracy and reliability need to be established before using them as a modality to study sexual dimorphism. Multidetector CT (MDCT) of MS has already proven to be valuable in studying sexual dimorphism [4]. Radiography is employed in forensic pathology for human identification, especially when bodies are decomposed, fragmented, or burned [5]. Sinus radiography, ranging from conventional methods like Water's view to advanced technologies such as computed tomography (CT) and cone beam computed tomography (CBCT) [6] has been employed for identifying skeletal remains and determining gender. Other methods such as MDCT [4], sinus endoscopy [7], and lateral encephalogram are also helpful for identification in the forensic field. Sinus endoscopy as a part of surgery or diagnosis improves visualization and allows for better preservation of normal structures thus useful for identification in forensic science [7].

Lateral cephalogram is particularly valuable because it provides detailed architectural and morphological information about the skull, offering additional characteristics and multiple points of comparison [6,8]. Researchers have found this conventional radiograph to be cost-effective, widely available, and reliable, with high accuracy rates for determining sexual dimorphism [9]. Various authors have explored the discriminative potential of maximum height, width, or a combination of both of MS using lateral cephalograms [5,6]. However, to our knowledge, such methodology has not yet been employed as a discriminant function to study sexual dimorphism in the North Indian population. Given this context, the present study was conducted to evaluate the accuracy and reliability of using MS morphometric parameters for gender determination in the North Indian population, to fill the gap in region-specific forensic data and enhance the precision of gender determination methods applicable to North Indian demographics.

## Materials and methods

This retrospective study was conducted in the department of forensic medicine and toxicology along with the dentistry department wherein a minimum of 74 consecutive lateral cephalograms were pulled out from the database and studied. The study specifically focused on the North Indian population, with samples drawn from the region of Punjab (37 samples), Haryana (22 samples), and Rajasthan (15 samples). Participants were selected to represent the ethnic diversity within this region. The study was approved by the Research Advisory Committee.

Inclusion criteria

Lateral cephalograms of patients of known sex and age (more than 18 years). Adults were included in the study because the dimensions of the MS stabilize after the second decade of life; by this age, the MS reaches its final position.

Exclusion criteria

Poor quality of lateral cephalogram, cases of facial trauma, MS fracture, congenital developmental disorders, and any pathology or any bony pathology were excluded from the study.

Study methodology

**Figure 1:**
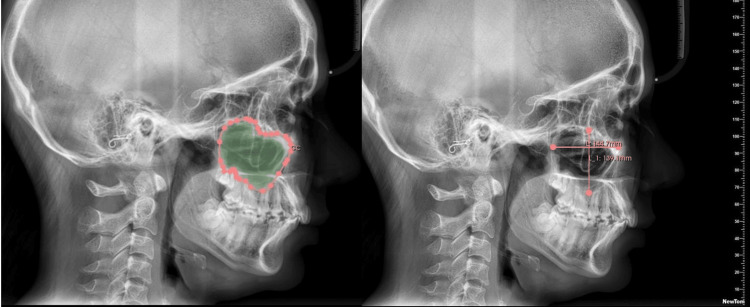
The measurement of the width and the height of the MS was also done using the same software. The width of the MS was defined as the widest distance which was measured on the horizontal line from the anterior wall (A) to the posterior wall of the MS (B). The height of the MS was defined as the longest distance drawn as a vertical line from the cranial wall (C) to the caudal wall of the MS (D) MS: maxillary sinus

About 74 lateral cephalograms (37 males and 37 females) of the adult age group (aged >18 years) were selected from the database available in the dental department after inclusion and exclusion criteria were satisfied. The height (mm), width (mm), and surface area (mm^2^) of the MS were measured using the NewTom CBCT machine (NewTom, Imola, Italy) with Slicer software. Magnification correction was done using a calibration device on the lateral cephalogram before taking the measurements. The surface area was calculated by tracing and marking the boundaries of MS using tools present in the software called 3D Slicer, version Slicer 5.6.1 (2023). The measurement of the width and the height of the MS was also done using the same software. The width of the MS was defined as the widest distance which was measured on the horizontal line from the anterior wall (A) to the posterior wall of the MS (B). The height of the MS was defined as the longest distance drawn as a vertical line from the cranial wall (C) to the caudal wall of the MS (D) (Figure 1).

Statistical analysis

Data was analyzed using IBM SPSS Statistics for Windows, Version 29 (Released 2021; IBM Corp., Armonk, New York, United States). Discriminant function analysis (linear discriminant analysis (LDA) model) was performed for the determination of gender. The sensitivity and specificity of the gender discriminants were also determined. An independent t-test was done to evaluate the difference between the gender and the volume of the sinuses. Pearson’s t-test was done to find any possible correlation between age and sinus area.

## Results

The descriptive statistics for age, height, width, and surface area, for females and males present a detailed comparative analysis based on several key metrics. Both groups had 37 valid observations for each variable, allowing for a robust comparison. There was no regional variation among samples taken from Punjab, Haryana, and Rajasthan.

The mean age of females was 23.86 years, with a standard error of 1.69 years. Males had a slightly lower mean age of 20.75 years and a standard error of 1.45 years. Table 1 shows the value of the height, width, and surface area of the MS.

**Table 1:** Values of the height, width, and surface area of the maxillary sinus SE: standard error; CV: coefficient of variations; MS: maxillary sinus

Measured parameters	Values (mean±SE)		
	Male	Female	p-value
Height of MS (in mm)	139.23±6.00 (CV=0.26)	133.76±3.35 (CV=0.15)	<0.01
Width of MS (in mm)	125.60±5.03 (CV=0.24)	121.56±3.72 (CV=0.18)	<0.01
Surface area of MS (in mm^2^)	18,71.82±3,371.70 (CV=1.09)	13,210.40±713.46 (CV=0.32)	<0.01

The comparative analysis thus revealed that while females and males had distinct average values for age in years, height (mm), width (mm), and surface area (mm^2^), males exhibited greater variability in their measurements across all variables. This variability was supported by higher standard errors and coefficients of the LDA model demonstrated high accuracy and robust performance metrics, effectively classifying female and male observations. Surface area emerged as the most influential feature, followed by age. Despite deviations from multivariate normality, the model maintained strong discriminative power, as evidenced by its high accuracy, area under the curve (AUC) variation, and broader confidence intervals.

Spearman's correlation analysis

**Figure 2:**
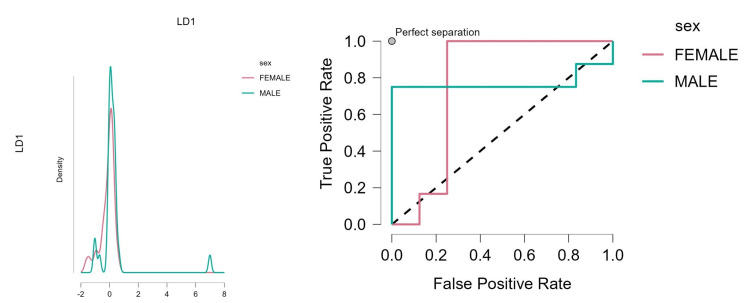
The LDA model achieved an accuracy of 0.85 for both classes, indicating a high level of correct predictions, and in the ROC plot, the area under the curve (AUC) was 0.77, suggesting good discriminative ability LDA: linear discriminant analysis; ROC: receiver operating characteristic

The Spearman's correlation analysis revealed several significant relationships among the variables. Age negatively correlates with surface area (rho=-0.271, p=0.019) and height (rho=-0.300, p=0.009), indicating that as age increases, both surface area and height tend to decrease. Surface area positively correlates with height (rho=0.513, p<0.001) and width (rho=0.420, p<0.001), suggesting that larger surface areas are associated with greater heights and widths. Height also positively correlates with width (rho=0.557, p<0.001). These findings provide valuable insights into structural characteristics and their interdependencies. The LDA model achieved an accuracy of 0.85 for both classes, indicating a high level of correct predictions, and in the receiver operating characteristic (ROC) plot, the AUC was 0.77, suggesting good discriminative ability (Figure 2).

The LDA model demonstrated high accuracy and robust performance metrics, effectively classifying female and male observations. Surface area emerged as the most influential feature, followed by age. Despite deviations from multivariate normality, the model maintained strong discriminative power, as evidenced by its high accuracy and AUC.

## Discussion

The clinical importance and significance of lateral cephalograms in gender determination are substantial. Lateral cephalograms provide detailed images of craniofacial structures, which exhibit sexually dimorphic traits. These radiographic images are non-invasive and widely used in dental and orthodontic practices, making them a convenient tool for gender identification [10]. Overall, the use of lateral cephalograms for gender determination is clinically significant as it integrates seamlessly into existing medical practices, providing a reliable, non-invasive, and accessible method for distinguishing between male and female craniofacial structures.

To date, a discriminant function specifically tailored for determining sex in the North Indian population has not been established [5]. Given that sexual differentiation varies across different races, findings applicable to one group may not hold true for another. Consequently, the discriminant function technique was employed to assess sex determination in skulls from various adolescents to establish population-specific standards [10,11]. This study represents a novel approach, aiming to evaluate the role of cephalometry in gender determination, focusing on its accuracy and reliability.

Hsiao et al. developed a method using lateral radiographic cephalometry and discriminant function analysis to determine sex, achieving 100% accuracy in the Taiwanese adult population [11]. Patil and Mody utilized a discriminant function based on 10 cephalometric variables, achieving 99% accuracy in sex determination [10]. Veyre-Goulet et al. demonstrated that sex could be determined with 95.6% accuracy using a discriminant function derived from various cephalometric measurements on adult dry skulls in the European population [12]. Variations in sinus dimensions can be influenced by genetic and environmental factors unique to different populations. By analyzing the MS morphometry in North Indians, this study provides tailored data that can improve the accuracy of forensic identification processes in this demographic. Additionally, using the MS as a reliable anatomical marker for gender determination can practically enhance the capabilities of forensic science in resolving mass disaster and crime scene investigations in North India.

Radiographic images provide sufficient measurements for MSs that are otherwise inaccessible [13]. Due to these developmental stages, only cephalograms of adults (aged 18 years and older) were included in the study.

As the age advances, the volume of the MS decreases in both genders [14,15]. This is attributed to the mineral loss in the bony walls of the air sinuses [13]. In the present study, age negatively correlates with surface area (rho=-0.271, p=0.019) and maximum height (rho=-0.300, p=0.009), indicating that as age increases, both surface area and maximum height tend to decrease.

In the year 2004, a similar study was conducted by Fernandes to determine gender from MS. Measurements of their study revealed that males had larger MSs than females, with an accuracy rate of 79.0% [16]. Teke et al. found accuracy rates of 69.4% for females and 69.2% for males [17]. Uthman et al. determined that 74.4% of male sinuses and 73.3% of female sinuses were correctly sexed, with an overall accuracy of 73.9% [18]. Vidya et al. examined the height, length, width, and volume of MSs in 30 dry skulls of South Indian origin and reported slightly larger measurements and volumes for males [19]. Amin and Hassan conducted a study using multi‑detector CT (MDCT) scan, eight MS measurements were assessed in 96 adult non‑pathologic Egyptians. The study concluded that the correct predictive accuracy was 70.8% in males and 62.5% in females [20]. Kanthem et al. noted that the dimensions and volumes of MSs on both sides were significantly larger in males compared to females when using CT [3]. The results of these studies are closely aligned with the findings of the present study.

It has been reported that ethnicity and environmental factors can affect the sizes of MSs [8]. Fernandes [16] conducted a study on 53 dried skulls, comprising 13 European males, 13 European females, 13 Zulu males, and 14 Zulu females. They used neural networks and linear measurements to estimate the volume of the MS. The study found significant ethnic and gender variations. Specifically, European crania had significantly larger antral volumes compared to Zulu crania, and males had larger volumes than females. The dimensions of European sinuses were larger than those of Zulu sinuses. The medial antral wall of the sinus was key in ethnic classification. In the current study, the morphometric parameters of the MS in individuals of North Indian origin were found to be smaller than those in their European counterparts. Recognizing these variations can enhance the accuracy of forensic Identification. The knowledge of ethnic differences in MS dimensions can aid forensic experts in the identification of individuals based on skeletal remains, contributing to more precise determinations of ethnicity. The discriminant analysis achieved a 90% success rate in predicting ethnicity and a 79% success rate in predicting gender [16].

In summary, the present study highlights that while females and males have distinct average values for age, surface area, maximum height, and maximum width, males exhibit significantly greater variability in these measurements. This greater variability is consistently observed through higher standard errors, coefficients of variation, and broader confidence intervals for males. These findings are corroborated by previous research, reinforcing the conclusion that males tend to have more diverse physical characteristics compared to females. This greater variability in males has important implications for studies involving physical measurements and should be considered in research and clinical practice

Limitations

The limitations of this study include a relatively small sample size, which may affect the generalizability of the results. Additionally, the study only included adult participants aged 18 and above, potentially excluding relevant developmental variations in younger individuals. The reliance on lateral cephalograms, a two-dimensional imaging technique, may not capture the full complexity of the MS anatomy compared to three-dimensional methods like CT or CBCT. Furthermore, variations in radiographic quality and positioning could introduce measurement errors. Last, the study did not account for potential ethnic or genetic differences that could influence sinus dimensions.

## Conclusions

The clinical importance of this study lies in its potential to enhance gender determination in forensic and anthropological contexts, given the significant difference in MS surface area between males and females. The statistically significant findings and good discriminative ability highlight its utility as a reliable anatomical marker. The novelty of this study is underscored by its focus on the MS surface area, an underexplored metric, providing new insights into gender differentiation. These results could improve the accuracy of forensic identification and offer a new dimension for anatomical and anthropological research.

## References

[1] Anon JB, Klimek L, Mosges R, Zinreich SJ (1997). Computer-assisted endoscopic sinus surgery. An international review. Otolaryngol Clin North Am.

[2] Kim S, Ward LA, Butaric LN, Maddux SD (2022). Human maxillary sinus size, shape, and surface area: Implications for structural and functional hypotheses. Am J Biol Anthropol.

[3] Kanthem RK, Guttikonda VR, Yeluri S, Kumari G (2015). Sex determination using maxillary sinus. J Forensic Dent Sci.

[4] Attia AM, El-Badrawy AM, Shebel HM (2012). Gender identification from maxillary sinus using multi-detector computed tomography. Mansoura J Forensic Med Clin Toxicol.

[5] Sidhu R, Chandra S, Devi P, Taneja N, Sah K, Kaur N (2014). Forensic importance of maxillary sinus in gender determination: a morphometric analysis from Western Uttar Pradesh, India. Eur J Gen Dent.

[6] Mathur RU, Mahajan AM, Dandekar RC, Patil RB (2014). Determination of sex using discriminant function analysis in young adults of Nashik: a lateral cephalometric study. J Adv Med Dent Sci Res.

[7] Sindel A, Turhan M, Ogut E, Akdag M, Bostancı A, Sindel M (2014). An endoscopic cadaveric study: accessory maxillary ostia. Dicle Med J.

[8] Kim GR (1962). A morphological study of the paranasal sinuses in Koreans. Yonsei Med J.

[9] Kiruba LN, Gupta C, Kumar S, D’Souza D’Souza (2014). A: A study of morphometric evaluation of the maxillary sinuses in normal subjects using computer tomography images. Arch Med Health Sci.

[10] Patil KR, Mody RN (2005). Determination of sex by discriminant function analysis and stature by regression analysis: a lateral cephalometric study. Forensic Sci Int.

[11] Hsiao TH, Tsai SM, Chou ST, Pan JY, Tseng YC, Chang HP, Chen HS (2010). Sex determination using discriminant function analysis in children and adolescents: a lateral cephalometric study. Int J Legal Med.

[12] Veyre-Goulet SA, Mercier C, Robin O, Guérin C (2008). Recent human sexual dimorphism study using cephalometric plots on lateral teleradiography and discriminant function analysis. J Forensic Sci.

[13] Sahlstrand-Johnson P, Jannert M, Strömbeck A, Abul-Kasim K (2011). Computed tomography measurements of different dimensions of maxillary and frontal sinuses. BMC Med Imaging.

[14] Jasim HH, Al-Taei JA (2013). Computed tomographic measurement of maxillary sinus volume and dimension in correlation to the age and gender (comparative study among individuals with dentate and edentulous maxilla). J Baghdad Coll Dent.

[15] Nunes Rocha MF, Dietrichkeit Pereira JG, Alves da Silva RH (2021). Sex estimation by maxillary sinus using computed tomography: a systematic review. J Forensic Odontostomatol.

[16] Fernandes CL (2004). Forensic ethnic identification of crania: the role of the maxillary sinus - a new approach. Am J Forensic Med Pathol.

[17] Teke HY, Duran S, Canturk N, Canturk G (2007). Determination of gender by measuring the size of the maxillary sinuses in computerized tomography scans. Surg Radiol Anat.

[18] Uthman AT, Al-Rawi NH, Al-Naaimi AS, Al-Timimi JF (2011). Evaluation of maxillary sinus dimensions in gender determination using helical CT scanning. J Forensic Sci.

[19] Vidya CS, Shamasundar NM, Manjunatha B, Raichurkar K (2013). Evaluation of size and volume of maxillary sinus to determine gender by 3D computerized tomography scan method using dry skulls of South Indian origin. Int J Curr Res Rev.

[20] Amin MF, Hassan EI (2012). Sex identification in Egyptian population using multidetector computed tomography of the maxillary sinus. J Forensic Leg Med.

